# Characterization of Staphylococci and Streptococci Isolated from Milk of Bovides with Mastitis in Egypt

**DOI:** 10.3390/pathogens9050381

**Published:** 2020-05-15

**Authors:** Wedad Ahmed, Heinrich Neubauer, Herbert Tomaso, Fatma Ibrahim El Hofy, Stefan Monecke, Ashraf Awad Abdeltawab, Helmut Hotzel

**Affiliations:** 1 Friedrich-Loeffler-Institut, Institute of Bacterial Infections and Zoonoses, Naumburger Str. 96a, 07743 Jena, Germany; wedad.ahmed@fvtm.bu.edu.eg (W.A.); heinrich.neubauer@fli.de (H.N.); Herbert.Tomaso@fli.de (H.T.); 2 Department of Microbiology, Faculty of Veterinary Medicine, Benha University, PO Box 13736 Moshtohor Toukh, Egypt; fatmaelhofy@yahoo.com (F.I.E.H.); ashraf.awad@fvtm.bu.edu.eg (A.A.A.); 3 Leibniz Institute of Photonic Technology (IPHT), Albert-Einstein-Str. 9, 07745 Jena, Germany; stefan.monecke@leibniz-ipht.de; 4 InfectoGnostics Research Campus Jena e. V., Philosophenweg 7, 07743 Jena, Germany

**Keywords:** *Staphylococcus*, *Streptococcus*, resistance gene, DNA microarray, mastitis, Egypt

## Abstract

The aim of this study was to characterize staphylococci and streptococci in milk from Egyptian bovides. In total, 50 milk samples were collected from localities in the Nile Delta region of Egypt. Isolates were cultivated, identified using matrix-assisted laser desorption/ionization time-of-flight mass spectrometry (MALDI-TOF MS), and antibiotic susceptibility testing was performed by the broth microdilution method. PCR amplifications were carried out, targeting resistance-associated genes. Thirty-eight *Staphylococcus* isolates and six *Streptococcus* isolates could be cultivated. *Staphylococcus aureus* isolates revealed a high resistance rate to penicillin, ampicillin, clindamycin, and erythromycin. The *mec*A gene defining methicillin-resistant *Staphylococcus aureus*, *erm*(C) and *aac-aph*D genes was found in 87.5% of each. Coagulase-negative staphylococci showed a high prevalence of *mec*A, *bla*Z and *tet*K genes. Other resistance-associated genes were found. All *Streptococcus dysgalactiae* isolates carried *bla*Z, *erm*(A), *erm*(B), *erm*(C) and *lnu*A genes, while *Streptococcus suis* harbored *erm*(C), *aph*A-3*, tet*L and *tet*M genes, additionally. In *Streptococcus gallolyticus*, most of these genes were found. The *Streptococcus agalactiae* isolate harbored *bla*Z, *erm*(B), *erm*(C), *lnu*A, *tet*K, *tet*L and *tet*M genes. *Streptococcus* *agalactiae* isolate was analyzed by DNA microarray analysis. It was determined as sequence type 14, belonging to clonal complex 19 and represented capsule type VI. Pilus and cell wall protein genes, *pav*A, *cad*D and *emr*B/*qac*A genes were identified by microarray analysis.

## 1. Introduction

Mastitis is an infection or inflammation of the mammary gland. It is the most common bacterial disease on dairy farms and leads to a reduction in milk production, with high economic losses due to high costs of treatment and the need of disposing of potentially contaminated milk. The prevention and treatment of mastitis lead to the administration of a considerable amount of antimicrobials to adult dairy cattle [1]. Microorganisms like *Escherichia* (*E*.) *coli*, different *Staphylococcus* (*S*.) and *Streptococcus* (*St*.) species are transmitted through colostrum to young calves and can cause gastrointestinal and pulmonary diseases. In some cases, this leads to the death of calves [2].

Inflammation of the udder in the case of mastitis is due to the release of leukocytes into the mammary gland in response to the invasion of the teat canal by microbes, their multiplication, and the production of toxins which all cause injury to milk-secreting tissue and to the various ducts within the mammary gland. The process results in a reduction of the amount of milk and in a change of the milk composition, with a high level of leukocytes or somatic cells [3].

Mastitis-diseased cattle can transmit pathogenic bacteria to humans through milk consumption, thus, they can be regarded as a public health hazard. Diseases that have been shown previously to be transmissible by milk from livestock to humans include tuberculosis, brucellosis, diphtheria, scarlet fever, streptococcal sore throat, and Q fever. Pasteurization techniques can control these diseases, but several bacteria still contribute to illness and disease outbreaks [4]. There are two types of mastitis, categorized into contagious and environmental mastitis, and both can be caused by a wide range of microorganisms [5]. Contagious pathogens are those for which the udders of infected cattle act as the main reservoir. These microorganisms can spread from cow to cow during milking, resulting in chronic subclinical infections. They include *S. aureus*, *St. agalactiae*, *Mycoplasma* species and *Corynebacterium bovis* [6]. Environmental mastitis is an intra-mammary infection caused by pathogens which are mainly present in the environment of cattle [7]. The majority of infections are clinical and of short duration [8]. Environmental pathogens include *E. coli*, *Klebsiella* species, *St. dysgalactiae* and *St. uberis*.

*S. aureus* is a coagulase-positive and Gram-positive bacterium, which is among the main etiological pathogens of contagious bovine mastitis [9]. This microorganism is well known for its high resistance to a wide range of antimicrobial agents and its ability to persist in bovine mammary epithelial cells, which allows it to evade the host immune system and to survive inside a wide variety of mammalian cells. This ability also aggravates antimicrobial therapy [10]. *S. aureus* is a human pathogen causing a variety of diseases like skin and soft tissue infections, but also food intoxications. Recently, coagulase-negative staphylococci (CoNS) have become the most common agents causing bovine mastitis. They are now predominant over *S. aureus* and have been considered as emerging mastitis pathogens. Species such as *S. sciuri, S. haemolyticus, S. chromogenes, S. epidermidis, S. saprophyticus,* and *S. simulans* belong to the CoNS group [11]. Some of the species are human pathogens.

*St. agalactiae*, referred to as group B *Streptococcus* (GBS) is an important pathogen in humans and a range of animal species. It is a common cause of mastitis in dairy cattle [12]. *St. agalactiae* is, in contrast to *S. aureus*, one of the mastitis-causing pathogens that can only grow and multiply in the udder. However, it can survive for short time periods on hands, parts of milking machines and teat skin, leading to its spread from cow to cow during milking. *St. agalactiae* is most commonly introduced into a clean herd when an infected cow is purchased. Because of the silent nature of infection and its highly contagious nature, infections can spread quickly. In humans, GBS causes serious neonatal infections, invasive diseases and other infections in adults, especially in the elderly. Other *Streptococcus* species show a zoonotic potential, too.

Antimicrobials are commonly used for the prevention and control of mastitis. Unfortunately, the therapeutic result is limited, due to the antimicrobial resistance of pathogens [13]. The emergence of multidrug-resistant bacteria has become a major threat to animal and human health [14]. This problem may not only limit the option for effective treatment, but also the spreading of resistance genes from contaminated milk to human normal flora [15].

The identification and characterization of staphylococci and streptococci can be performed by biochemical investigations, molecular assays (PCR and DNA sequencing) and physical techniques like matrix-assisted laser desorption/ionization time-of-flight mass spectrometry (MALDI-TOF MS). In recent years, DNA microarrays have been developed not only to investigate the expression of multiple genes in tissues but also for the genotyping of bacteria. DNA microarray technology allows the simultaneous detection of a high number of molecular targets. This approach facilitates a genotype-based assessment of virulence, as well as of the antibiotic resistance of a given isolate [16].

Here, a description is given which reflects what the Egyptian farmers and consumers expect when drinking milk directly from producers without pasteurization. The prevalence of staphylococci and streptococci in milk from cattle and buffaloes and the characterization of isolates is described concerning their phenotypic and genotypic resistance to antibiotics, because the knowledge about the situation in Egypt is limited.

## 2. Materials and Methods

### 2.1. Sample Collection and Cultivation

The present study was conducted in 2018 and 2019 on 50 milk samples of dairy animals from 50 different localities in Qalyubia and Monufia governorates in the Nile Delta region of Egypt. All dairy cattle and buffaloes were local Egyptian breed and kept by smallholders (1–5 animals) at different localities. They were hand-milked twice daily.

All animals were subjected to clinical examination. Animals with clinical mastitis were identified when one or more of the following signs were observed: cardinal signs of inflammation in one or more of udder’s quarters, signs of systemic reaction, such as fever, depression, disturbed appetite and abnormal physical character of milk such as clot formation, discoloration, altered viscosity, aberrant smell or presence of blood. Due to the absence of observable clinical signs in animals with subclinical mastitis, the presumptive diagnosis was done based on laboratory diagnostic tests of milk samples, including the California mastitis test (CMT).

Milk samples were taken after washing and drying of the udder. Teat ends were disinfected with cotton swabs soaked in 70% ethanol. The first few streams were discarded. Approximately 10 mL of milk from each udder quarter were put into sterile tubes. Samples were transported to the laboratory on ice and stored at 4 °C for subsequent bacteriological analyses according to National Mastitis Council guidelines [17].

Isolation of bacteria from milk samples was carried out as described by the National Mastitis Council [17]. A loopful of milk sample was streaked on blood agar (Oxoid Deutschland GmbH, Wesel, Germany), supplemented with 5% sheep red blood cells and then subcultured on selective media: Mannitol Salt Agar, Edwards Medium and Brilliance ESBL Agar (Oxoid Deutschland GmbH), for the identification of expanded-spectrum beta-lactamase (ESBL)-producing microorganisms. All plates were incubated aerobically at 37 °C for 24 h. The plates were examined for colony morphology, pigmentation and hemolytic characteristics after 24–48 h.

### 2.2. MALDI-TOF MS

Isolates were identified using MALDI-TOF MS [18]. Briefly, bacteria from overnight cultures were suspended in 300 µL of bi-distilled water and mixed with 900 µL of ethanol (96% vol/vol; Carl Roth GmbH, Karlsruhe, Germany) for precipitation. After centrifugation for 5 min at 10,000× *g*, the supernatant was removed and the pellet was re-suspended in 50 µL of 70% (vol/vol) formic acid (Sigma-Aldrich Chemie GmbH, Steinheim, Germany). Fifty microliters of acetonitrile (Carl Roth GmbH, Karlsruhe, Germany) were added, mixed and centrifuged for 5 min at 10,000× *g*. One and a half microliters of the supernatant were transferred onto an MTP 384 Target Plate Polished Steel TF (Bruker Daltonik GmbH, Bremen, Germany). After air-drying the material was overlaid with 2 µL of a saturated solution of α-cyano-4-hydroxycinnamic acid (Sigma-Aldrich Chemie GmbH, Steinheim, Germany) in a mix of 50% acetonitrile and 2.5% trifluoroacetic acid (Sigma-Aldrich Chemie GmbH, Steinheim, Germany). After air-drying spectra were acquired with an Ultraflex instrument (Bruker Daltonik GmbH, Bremen, Germany), the instrument was calibrated with the IVD Bacterial Test Standard (Bruker Daltonik GmbH, Bremen, Germany). An analysis was carried out with the Biotyper 3.1 software (Bruker Daltonik GmbH, Bremen, Germany). An interpretation of results was performed according to the manufacturer’s recommendation: score of ≥ 2.3 represented reliable species-level identification; score 2.0–2.29, probable species-level identification; score 1.7–1.9, probable genus-level identification, and score ≤ 1.7 was considered an unreliable identification.

### 2.3. DNA Extraction

Genomic bacterial DNA was prepared from colonies with typical growth and subculture on blood agar. A loop-full of bacteria was added to 0.2 mL aliquot of lysis enhancer A2 dissolved in lysis buffer A1 (both from the StaphyType Kit, Alere Technologies GmbH, Jena, Germany), followed by incubation for 60 min at 37 °C and 550 rpm in a thermomixer.

For staphylococci, 10 µL of lysostaphin (Sigma-Aldrich Chemie GmbH; 2 mg/mL bidistilled water) and 5 µL of lysozyme (10 mg/mL bidistilled water) were used for lysis and incubated at 37 °C and 550 rpm in a thermomixer.

For streptococci, 10 µL of achromopeptidase (Sigma-Aldrich Chemie GmbH; 100 units dissolved in 5 mL of phosphate-buffered saline (PBS) and stored frozen in small aliquots) and 5 µL of lysozyme (10 mg/mL bidistilled water) were used for lysis and incubated at 37 °C and 550 rpm in a thermomixer.

After lysis, the samples were processed using the High Pure PCR Template Purification Kit (Roche Diagnostics, Mannheim, Germany), according to the instructions of the manufacturer.

### 2.4. Antibiotic Susceptibility Testing

The antibiotic susceptibility testing was performed by broth microdilution method with the MICRONAUT system for Gram-positive bacteria using commercial 96-well microtiter plates (MICRONAUT-S MRSA/GP; Merlin, Bornheim, Germany), according to the manufacturer’s recommendations. MICRONAUT system for Gram-positive bacteria allowed the determination of minimum inhibitory concentrations (MICs) of 22 antimicrobial agents, including ampicillin (β-lactam), cefoxitin (β-lactam; cephamycin), ceftaroline (cephalosporin 5th generation), clindamycin (lincosamide), daptomycin (cyclic lipopeptide), erythromycin (macrolide), erythromycin/clindamycin, fosfomycin (epoxide antibiotic), fusidic acid (steroid antibiotic), gentamicin (aminoglycoside), linezolid (oxazolidinone), moxifloxacin (fluorchinolone 4th generation), mupirocin, oxacillin (β-lactam), penicillin G (β-lactam), rifampicin (ansamycine), synercid (streptogramin), teicoplanin (glycopeptide), tigecycline (glycylcycline), trimethoprim/sulphamethoxazole (trimethoxy-benzyl pyrimidine/sulfonamide), and vancomycin (glycopeptide).

### 2.5. Detection of Resistance-Associated Genes

For staphylococci, PCR amplifications were carried out, targeting resistance-associated genes of β-lactam antibiotics (*bla*Z, *mec*A, *mec*B, *mec*C), tetracyclines (*tet*K, *tet*L, *tet*M, *tet*S, *tet*O), erythromycin/clindamycin (*erm*(A), *erm*(B), *erm*(C)), macrolides (*msr*C), aminoglycosides (*aac-aph*D), vancomycin (*van*A, *van*B, *van*C1) and linezolid (*optr*A, *val*S, *cfr*).

For streptococci, PCR amplifications were done to detect the genes responsible for resistance to lincosamide (*lnu*A and *lnu*D), macrolides (*mef*A, *erm*(A), *erm*(B), *erm*(C) and *erm*(TR)), penicillin (*bla*Z), aminoglycosides (*aad*-6, *aph*A-3, *aac*6-*aph*2) and tetracycline (*tet*K, *tet*L, *tet*M, *tet*S and *tet*O). PCR conditions followed those given in the references in Table 1. PCR products were analyzed by gel electrophoresis on 2% agarose gels following staining with ethidium bromide and visualized under UV light.

### 2.6. Microarray Analysis

For the analysis of streptococci, a microarray specifically developed and validated for *St. agalactiae* (Alere Technologies GmbH, Jena, Germany) was used, targeting group B streptococci virulence-associated markers and resistance-associated genes. Additionally, macrolide/lincosamide, tetracycline, and heavy metal resistance genes, genes associated with phages and gene motility were included. Protocols, data interpretation, and evaluation have been described previously [36]. Briefly, a linear and thermally synchronized primer elongation reaction was used for labeling. A mix of 1 to 2 µg of unfragmented RNA-free target DNA, 1.5 µL of a primer mixture (0.135 µmol/L each), dNTP mix, *Taq* DNA polymerase, and biotin-16-dUTP was amplified and labeled, using the following program: an initial denaturation at 96 °C for 5 min was followed by 55 cycles (60 s at 96 °C, 20 s at 50 °C, and 40 s at 72 °C). After washing, the hybridization of labeled DNA samples in ArrayStrips (Alere Technologies GmbH, Jena, Germany) was carried out. After washing, the microarrays were incubated with 100 µL of horseradish peroxidase-streptavidin mixture for 15 min. A repeated washing step followed. Finally, 100 µL of a precipitating substrate were added. After 5 min of incubation at room temperature (without shaking), the substrate was removed and the arrays were scanned and analyzed using the ArrayMate reader (Alere Technologies GmbH, Jena, Germany), using a specific software.

## 3. Results

### 3.1. Bacterial Isolation and Identification by MALDI-TOF MS

In this study, 38 *Staphylococcus* isolates were obtained from 50 milk samples of cattle and buffaloes. Additionally, six *Streptococcus* isolates could be cultivated. Identification by MALDI-TOF MS resulted in *S. warneri* (*n* = 9), *S. aureus* (*n* = 8), *S. pasteuri* (*n* = 8), *S. xylosus* (*n* = 4), *S. epidermidis* (*n* = 2), *S. chromogenes* (*n* = 2), *S. cohnii* (*n* = 1), *S. hyicus* (*n* = 1), *S. haemolyticus* (*n* = 1), *S. sciuri* (*n* = 1), *S. lentus* (*n* = 1), *St. dysgalactiae* (*n* = 3), *St. agalactiae* (*n* = 1; 19CS0081), *St. gallolyticus* (*n* = 1) and *St. suis* (*n* = 1). The distribution of isolates from cattle and buffaloes is given in Table 2 and Table 3.

In some milk samples more than one pathogen was detected. A few of them harbored two or three different *Staphylococcus* species. Additionally, mixed infections with *Staphylococcus* and *Streptococcus* species occurred.

### 3.2. Antimicrobial Susceptibility Profiles of Staphylococci

All *Staphylococcus* isolates, 8 *S. aureus,* and 30 CoNS, were examined for their susceptibility to 22 antimicrobial agents. Table 4 shows that *S. aureus* isolates had high resistance rates to penicillin (87.5%), ampicillin, clindamycin, and erythromycin (75.0% each), respectively. All *S. aureus* isolates were fully susceptible to ceftaroline, teicoplanin, and vancomycin. Resistance rates to other antibiotics ranged between 25.0% and 62.5%.

Non-*Staphylococcus aureus* isolates showed high resistance rates to erythromycin, erythromycin/clindamycin, fosfomycin, fusidic acid, clindamycin, daptomycin, moxifloxacin and gentamicin, with 96.7%, 93.3%, 93.3%, 90.0%, 83.3%, 83.3%, 80.0% and 80.0%, respectively. Resistance rates of other antimicrobials ranged between 13.3% for vancomycin and mupirocin and 73.3% for penicillin, respectively.

With described microdilution plates and *Streptococcus* isolates, no valid results were obtained.

### 3.3. Detection of Resistance-associated Genes in Staphylococci

All *S. aureus* isolates harbored the *bla*Z gene associated with penicillin resistance, the *te*tK gene associated with tetracycline resistance and *val*S often found in the *optr*A operon connected with linezolid resistance (Table 5). Other frequently detected resistance determinants were *mec*A associated with β-lactam resistance, *erm*(C) for erythromycin resistance and *aac-aph*D responsible for aminoglycoside resistance in 87.5% of all isolates. The *erm*(B) (resistance to erythromycin) and *msr*C (macrolide resistance) genes were also found frequently, with 75.0%.

Non-*Staphylococcus aureus* isolates exhibited a high prevalence of resistance genes *mec*A, *bla*Z and *tet*K, with 96.6%, 80.0%, and 73.3%, respectively. Approximately half of the isolates harbored *aac-aph*D, *erm*(C) and *erm*(B) genes.

### 3.4. Detection of Resistance-Associated Genes in Streptococci

According to streptococci, *St. dysgalactiae* isolates (*n* = 3) showed the presence of the *bla*Z gene responsible for penicillin resistance in all isolates as well as *erm*(A), *erm*(B) and *erm*(C) associated with macrolide resistance and *lnu*A connected with lincosamide resistance (Table 6). Additionally, *tet*L and *tet*M genes associated with tetracycline resistance were found in these isolates. Additionally, the *aphA*-3 gene responsible for aminoglycoside resistance was detected in 2 isolates.

The *St. agalactiae* isolate harbored *bla*Z, *erm*(B), *erm*(C), *lnu*A, *tet*K, *tet*L and *tet*M genes as resistance-associated determinants, while *St. suis* isolate carried *bla*Z, *erm*(A), erm(B), *erm*(C), *lnu*A, *aph*A-3*, tet*L and *tet*M genes. The genes *bla*Z, *erm*(B), *erm*(C), *aph*A-3, *aad*-6, *lnu*A, *tet*K, *tet*L, *tet*M and *tet*S were detected in the *St. gallolyticus* isolate by PCR. The *aac*6-*aph*2 genes were not found in any of the *Streptococcus* isolates.

### 3.5. Microarray Analysis of Streptococcus Isolates

Six *Streptococcus* isolates of different species were tested with a microarray system. This system was developed and validated exclusively for *St. agalactiae* and only for this isolate (19CS0081) was a valid result obtained. The other *Streptococcus* isolates did not harbor more antibiotic resistance-associated genes, as detected by PCR investigation.

Isolate 19CS0081 was a *St. agalactiae* strain belonged to clonal complex (CC) 19. The sequence type (ST) was 14. The isolate represented capsule type VI, but it obtained a hybridization signal with one of the capsule III probes (cpsG-III), too. Belonging to the alpha antigenic cell wall protein genes, the *alp*-5 gene was detected. Pilus protein genes *pil*A1, *pil*B1 and *pil*C1 could be detected. From the group of surface proteins, the *sip* gene (surface immunogenic protein) was detected, as well as MSCRAMM (microbial surface components recognizing adhesive matrix molecules) adhesin protein gene *pav*A (fibronectin-binding protein gene). Besides the antimicrobial resistance-associated genes heavy metal resistance marker *cad*D encoding cadmium resistance protein D and multidrug resistance transporter genes, *emr*B/*qac*A were found in the *St. agalactiae* isolate.

## 4. Discussion

Staphylococci are major causative agents of clinical or subclinical bovine mastitis and generate important losses in the dairy industry in Egypt [37]. They are also considered as a risk factor for food poisoning in humans [38].

In this study, 38 *Staphylococcus* isolates were cultivated from 50 milk samples, whereas 32 came from cattle and buffaloes with clinical signs of mastitis and 18 from subclinical mastitis cases, respectively. The prevalence of staphylococci with 75.0% and 77.7% nearly agreed with the data of Dorgham et al. [39]. The authors detected staphylococci in 68.8% and 62.5% of milk samples from Egypt. The milk came from cattle, buffaloes, and goats with clinical and subclinical mastitis. Others also reported rates of 52.0% and 67.0% *Staphylococcus* positive milk samples, in cases of clinical and subclinical bovine mastitis in Egypt [40].

Eight isolates (16.0%) were identified as *S. aureus,* which was similar to other studies on mastitis milk samples in Egypt who reported about 16.1%, 14.9% and 25.8% *S. aureus* positive samples, respectively [41,42,43]. Other previous studies have confirmed that *S. aureus* and *St. agalactiae* were the most prevalent causative agents of mastitis in Egypt [44,45,46].

Several *Staphylococcus* species, like *S. warneri*, *S. pasteuri*, *S. xylosus,* and others were found in the milk samples of cattle and buffaloes. A similar spectrum of species was found in milk of cattle and buffaloes with subclinical mastitis, namely *S. intermedius*, *S. xylosus*, *S. epidermidis*, *S. hominis, S. sciuri*, *S. hyicus*, *S. lugdunensis* and *S. simulans* [47]. These reports complete the results of investigations made in cattle herds with mastitis problems, where *S. chromogenes, S. hyicus, S. simulans, S. epidermidis, S. hominis, S. haemolyticus, S. xylosus, S. warneri, S. sciuri, S. capitis, S. saprophyticus,* and *S. lentus* were found [48,49,50]. They assured that the environment was found as a reservoir, suggesting that intra-mammary infection with such bacterial species is possibly considered as an environmental hazard.

Streptococci form a large group of organisms. Some of them are associated with bovine udder infections. The most common pathogens causing bovine mastitis are *St. agalactiae, St. dysgalactiae* and *St. uberis* [51]. In the present study, in 12.0% of milk samples, *Streptococcus* isolates were detected, which was similar to results from China, where 8.7% of dairy cattle found positive for *Streptococcus* species [52]. Atypical streptococci found and connected with mastitis were *St. suis* and *St. gallolyticus*.

More than a single pathogen was detected in some of milk samples. There were cases with two or three different *Staphylococcus* species, as well as mixed infections of staphylococci and streptococci. This makes it difficult to identify the true mastitis causing agent. As a result, a possible antibiotic treatment is dependent on the antibiotic resistance of the different bacteria.

Seven out of eight isolates carried the *mec*A gene, which is defining them as methicillin-resistant *S. aureus* (MRSA). This is a high rate of MRSA from milk samples and this observation might indicate an uncontrolled usage of antibiotics. The results were supported by other investigations, which described the presence of *mec*A gene in phenotypic β-lactam-resistant *S. aureus* isolates in more than half of Egyptian milk samples originating from mastitis cases [53,54]. In contrast, an investigation on milk samples from Switzerland and Germany resulted in only 2 MRSA out of 128 isolates [55]. The presence of *mec*A gene in *S. aureus* isolates from bovine milk have been reported in previous studies from India [56], China [57], Italy [58], Tunisia [59] and Brazil [60]. MRSA isolates showed not only resistance to methicillin/oxacillin and other β-lactams; they were also resistant to other antibiotics as aminoglycosides, macrolides and/or quinolones [61]. In this study, all MRSA isolates showed multidrug resistance, which is in agreement with previous reports on MRSA isolated from dairy products [37,53,62,63]. The *mec*B and *mec*C genes also responsible for methicillin resistance were not detected. They were found only sporadically in *S. aureus* isolates from domestic animals yet, although in a Bavarian dairy herd, multiple cases of methicillin resistant CC130 *S. aureus* were found harboring the *mec*C gene [64].

Penicillin resistance of *Staphylococcus* species is usually mediated by *bla*Z gene about enzymatic hydrolysis of β-lactam ring of antibiotics. It was detected in all *S. aureus* isolates and 73.3% of non-*S. aureus* isolates, from which a high percentage showed phenotypic resistance to penicillin. High prevalence of *bla*Z gene in penicillin-resistant staphylococci was reported from Egypt [53], China [57] and Brazil [65]. Similar results were obtained with Polish *Streptococcus* isolates [66]. Resistance to penicillin G is very important, because this antimicrobial agent is the most recommended and used antibiotic for the treatment of staphylococcal mastitis. Increasing resistance to penicillin G can be explained by the uncontrolled use of antimicrobial agents in Egypt.

Resistance to macrolides (such as erythromycin) occurred among staphylococci in this study. Resistance to this group of antibiotics is conferred via a variety of mechanisms. Three related determinants, *erm* (A), *erm* (B), and *erm* (C) genes, have been identified to be mainly responsible for erythromycin resistance [67]. In this study, the three *erm* genes were detected in 25.0% to 87.5% of the *S. aureus* isolates. This is comparable to the results described previously [35]. Additionally, the macrolide resistance gene *msr*C was prevalent and the percentage in *S. aureus* was much higher than in other staphylococci. The *erm* (B) and *erm* (C) genes occurred in all *Streptococcus* isolates, which reflects the situation reported by other authors [68,69,70]. The main cause for this result may be due to the localization of these genes on transposons and the ability of a transfer from bacteria to another, including streptococci by horizontal gene transfer [68]. *Erm* (TR) gene was not detected in this study, which was in agreement with the results of Dogan et al. [71]. They described the absence of the *erm* (TR) gene in bovine streptococci, however, it could be detected in human isolates.

Aminoglycoside resistance (including gentamicin) was frequently detected in staphylococci and is caused, to a great extent, by the presence of *aac-aph*D genes, as previously described for Chinese isolates [57]. Aminoglycoside resistance in streptococci is mediated by *aad*-6 and *aph*A-3 genes, resulting in the enzymatic inactivation of antibiotics. Both genes were not detected in the *St. agalactiae* isolate, but at least one was present in other isolates, which is equal to results from Poland [66].

Tetracycline resistance genes spread among bacteria and can be found regularly in multidrug resistant bacteria [72]. In the current study, *tet*K, *tet*L, and *tet*M genes were frequently detected, which corresponded with reports from China for *S. aureus* [57] and isolates from Egypt for other CoNS [47]. The cause is the extensive and frequent usage of tetracycline in mastitis treatment and as prophylaxis to reduce bacterial infections in general in Egypt, which leads to increased resistance to this antimicrobial [73]. Tetracycline resistance-associated genes (*tet*K, *tet*L, *tet*M, and *tet*S) in streptococci were detected in all isolates, in which *St. gallolyticus* harbored all four genes. All isolates carried *tet*L and *tet*M genes, which was described equally for Polish isolates [66].

Linezolid is one of the few clinically effective drugs for the control of MRSA and MRCoNS infections. Resistance to linezolid in staphylococci is still very rare, but has been increased in recent years [74]. Transferable linezolid resistance due to the presence of the *cfr* gene has been known since the year 2000. The *cfr* gene was first discovered in a bovine *S. sciuri* isolate and was later reported in various *Staphylococcus* species. In this study, the *cfr* gene was not detected. The result was in agreement with a study on *Staphylococcus* isolates from chicken meat in Egypt [75].

Linezolid and phenicol resistance can be mediated by the *optr*A gene, too [76]. Phenotypic resistance to linezolid was detected in 50.0% of *S. aureus* and 70.0% of non-*S. aureus* isolates and the *optr*A gene was frequently detected by PCR. Previously, the presence of *optr*A gene connected with resistance to linezolid in *Staphylococcus* isolates was reported [77,78]. This result is alarming because linezolid is an antibiotic with good activity against MRSA, making it desirable for the treatment of staphylococcal infections [74].

Vancomycin resistance in staphylococci is very important, because vancomycin is becoming the final choice for the treatment of MRSA infections in humans [79]. Phenotypic vancomycin resistance in *S. aureus* was not determined [80], however, it was detected in other staphylococci, as described before [75]. In this study, the discrepancy between phenotypic resistance and the presence of resistance genes was noticeable. More than 60% of *S. aureus* isolates carried the *van*C1 gene, but did not show phenotypic resistance determined by the broth microdilution method. A similar situation arises with other staphylococci.

Although *erm* genes responsible for macrolide-lincosamide-type B streptogramin-associated resistance in *Streptococcus* species, lincosamide resistance was mediated by the presence of specific genes (*lnu*), which cause enzymatic inactivation of the drug due to nucleotide transferases. This mechanism was detected for the first time for *Enterococcus faecium* and thought to be exclusively present in this species, although later on, it was detected in other species like *St. agalactiae* and *St. uberis* [81,82]. In this study, the *lnu*A gene was detected in all streptococcal isolates, as it was identical with the results of a previous investigation [66].

Antibiotic resistance is increasing, because treatment of mastitis generally occurs by using antibiotics without testing of antibiotic susceptibilities of causative bacteria [83]. In this study, 75.0% of *S. aureus* isolates detected were multidrug-resistant and revealed a high phenotypic resistance to penicillin, ampicillin, and erythromycin/clindamycin, thus have been reported by [53] for *S. aureus* isolates and by [63], too. A similar situation was described for Egyptian CoNS isolates from cattle, buffaloes, and goats [39].

In this study, we used a microarray-based assay for the simultaneous detection of typing markers, resistance determinants and clinically relevant virulence factors in *St. agalactiae*. The system was developed especially for this species. For other *Streptococcus* species, only the probes for resistance determinants were useful, but gave no advantage in comparison to PCR assays. For *St. agalactiae*, it was possible to determine the ST and CC of the isolate. With DNA microarrays, it is possible to genotype isolates. The method is less cumbersome than MLST, in which an isolate is characterized after sequencing of seven house-keeping genes. Here, hybridization patterns for the *St. agalactiae* isolate appear to correspond to ST14 within CC19. CC19 is the most common but very diverse clonal complex. In a study about German *St. agalactiae*, 75% of human isolates and 32% of bovine isolates belonged to this CC [36]. The determined capsule type VI is a very rare type and was found in a German strain collection, only once in an isolate of human origin.

In Egyptian milk samples which were obtained from cattle and buffaloes of small farmers, several zoonotic bacteria like staphylococci, streptococci, *E. coli,* and enterococci were detected. Normally, the milk is directly consumed by farmers, sold to consumers or used for the production of soft cheese, etc., without pasteurization. The consumption of dairy products entails a health hazard, because some of the bacteria are able to infect humans or are causing agents of food poisoning. Reinforced is the risk by the increasingly occurred resistance of bacteria during the last decades.

In this study, multidrug-resistant isolates of different bacterial species were confirmed via phenotypic characterization as well as the detection of resistance determinants. One source of reinforced occurrence of resistant bacteria is to look for inexpertly and excessively use of antibiotics in veterinary medicine. Here, the consumption of antibiotics has to be reduced, veterinarians and farmers need to be trained in the use of antimicrobials, a diagnosis must be made before therapy, and last but not least, milk must be pasteurized. Additionally, governmental monitoring tools can help to reduce antibiotic usage.

## Figures and Tables

**Table 1:** Primers and their sequences used for the detection of antibiotic resistance-associated genes in *Staphylococcus* and *Streptococcus* isolates.

Antibiotic	Target Gene	Primer Sequences (5′-3′)	Expected Amplicon Size (bp)	Reference
Methicillin/ oxacillin	*mec*A	F: TCC AGA TTA CAA CTT CAC CAG G R: CCA CTT CAT ATC TTG TAA CG	161	[19]
	*mec*B	F: TTA ACA TAT ACA CCC GCT TG R: TAA AGT TCA TTA GGC ACC TCC	2263	[20]
	*mec*C	AL3: TCA AAT TGA GTT TTT CCA TTA TCA AL4: AAC TTG GTT ATT CAA AGA TGA CGA	1931	[20]
Penicillin	*bla*Z	F: ACT TCA ACA CCT GCT GCT GCT TTC R: TGA CCA CTT TTA TCA GCA ACC	172	[19]
	*bla*Z	F: AAG AGA TTT GCC TAT GCT TC R: GCT TGA CCA CTT TTA TCA GC	517	[21]
Vancomycin	*van*A	F: ATG AAT AGA ATA AAA GTT GCA ATA R: CCC CTT TAA CGC TAA TAC GAT CAA	1030	[22]
	*van*B	F: AAG CTA TGC AAG AAG CCA TG R: CCG ACA AAA TCA TCC TC	536	[22]
	*van*C1	F: GGA ATC AAG GAA ACC TC R: CTT CCG CCA TCA TAG CT	822	[23]
Erythromycin	*erm*(B)	F: GAA AAG GTA CTC AAC CAA ATA R: AGT AAC GGT ACT TAA ATT GTT TAC	639	[24]
	*erm*(A)	F: TAT CTT ATC GTT GAG AAG GGA TT R: CTA CAC TTG GCT TAG GAT GAA A	138	[19]
	*erm*(C)	F: CTT CTT GAT CAC GAT AAT TTC C R: ATC TTT TAG CAA ACC CGT ATT C	189	[19]
	*erm*(TR)	F: ATAGAAATTGGGTCAGGAAAAGG R: CCCTGTTTACCCATTTATAAACG	376	[25]
Macrolides	*msr*C	F: AAG GAA TCC TTC TCT CTC CG R: GTA AAC AAA ATC GTT CCC G	342	[26]
	*mef*A	F: AGT ATC ATT AAT CAC TAG TGC R: TTC TTC TGG TAC TAA AAG TGG	500	[25]
Tetracycline	*tet*K	F: TCG ATA GGA ACA GCA GTA R: CAG CAG ATC CTA CTC CTT	169	[27]
	*tet*L	F: TCG TTA GCG TGC TGT CAT R: GTA TCC CAC CAA TGT AGC CG	267	[27]
	*tet*M	F: GTG GAC AAA GGT ACA ACG AG R: CGG TAA AGT TCG TCA CAC AC	406	[27]
	*tet*O	F: AAC TTA GGC ATT CTG GCT CAC R: TCC CAC TGT TCC ATA TCG TCA	515	[27]
	*tet*S	F: TGG AAC GCC AGA GAG GTA TT R: ACA TAG ACA AGC CGT TGA CC	660	[28]
Aminoglyco-sides	*aac*6-*aph*2	F: CCA AGA GCA ATA AGG GCA TA R: CAC TAT CAT AAC CAC TAC CG	219	[29]
	*aac-aphD*	F: TAA TCC AAG AGC AAT AAG GGC R: GCC ACA CTA TCA TAA CCA CTA	227	[19]
	*aad-6*	F: AGA AGA TGT AAT AAT ATA G R: CTG TAA TCA CTG TTC CCG CCT	978	[30]
	*aphA-3*	F: GGG GTA CCT TTA AAT ACT GTA G R: TCT GGA TCC TAA AAC AAT TCA TCC	848	[31]
Linezolid, chlor-amphenicol	*optr*A	F: AGG TGG TCA GCG AAC TCA R: ATC AAC TGT TCC CAT TCA	1400	[32]
Linezolid	*val*S	F: GTA ACG ATC ATC ATT TGG G R: CTT TAT TAG AGC TCA ATG GGC	339	[33]
Oxazolidinone	*cfr*	F: TGA AGT ATA AAG CAG GTT GGG AGT CA R: ACC ATA TAA TTG ACC ACA AGC AGC	400	[32]
Lincosamide	*lnu*D	F: ACG GAG GGA TCA CAT GGT AA R: TCT CTC GCA TAA TAA CCT TAC GTC	475	[34]
	*lnu*A	F: GGT GGC TGG GGG GTA GAT GTA TTA ACT GG R: GCT CTC TTT GAA ATA CAT GGT ATT TTT CGA TC	323	[35]

**Table 2:** Prevalence of *Staphylococcus* and *Streptococcus* isolates in milk samples from cattle and buffaloes with clinical and subclinical mastitis.

Type of Mastitis	Origin of Milk	Number of Samples	*Staphylococcus aureus* Isolates		Non-*Staphylococ-* *cus aureus* Isolates		*Streptococcus* Isolates	
			No.	%	No.	%	No.	%
Clinical mastitis	Cattle	22	2	9.1	12	54.6	1	4.6
	Buffaloes	10	1	10.0	9	90.0	1	10.0
Subclinical mastitis	Cattle	5	1	20.0	5	100	1	20.0
	Buffaloes	13	4	30.8	4	30.8	3	23.1
Total		50	8	16.0	30	60.0	6	12.0

**Table 3:** Identified non-*Staphylococcus aureus* species recovered from 50 bovine milk samples.

CoNS	*S. warneri*	*S. pasteuri*	*S. xylosus*	*S. epidermidis*	*S. chromogenes*	*S. cohnii*	*S. hyicus*	*S. haemolyticus*	*S. sciuri*	*S. lentus*	Total
Cattle	3	5	3	2	2	0	0	0	1	1	17
Buffaloes	6	3	1	0	0	1	1	1	0	0	13

**Table 4:** Antimicrobial susceptibility of *Staphylococcus* isolates from milk.

Antibiotic	Class	*Staphylococcus aureus* Isolates (*n* = 8)				Non*-Staphylococcus aureus* Isolates (*n* = 30)			
		S	I	R	RR (%)	S	I	R	RR (%)
Ampicillin	β-Lactam	2	0	6	75.0	9	0	21	70.0
Cefoxitin	β-Lactam; cephamycin	4	0	4	50.0	15	3	12	40.0
Ceftaroline	Cephalosporin 5th generation	8	0	0	0.0	22	2	6	20.0
Clindamycin	Lincosamide	2	0	6	75.0	5	0	25	83.3
Daptomycin	Cyclic lipopeptide	2	1	5	62.5	3	2	25	83.3
Erythromycin	Macrolide	2	0	6	75.0	1	0	29	96.7
Erythromycin/ clindamycin		2	0	6	75.0	2	0	28	93.3
Fosfomycin	Epoxide antibiotic	6	0	2	25.0	1	1	28	93.3
Fusidic acid	Steroide antibiotic	4	0	4	50.0	2	1	27	90.0
Gentamicin	Aminoglyside	3	0	5	62.5	5	1	24	80.0
Gentamicin high level	Aminoglyside	3	0	5	62.5	16	2	12	40.0
Linezolid	Oxazolidinone	4	0	4	50.0	7	2	21	70.0
Moxifloxacin	Fluorchinolone 4th generation	4	0	4	50.0	5	1	24	80.0
Mupirocin		5	1	2	25.0	21	5	4	13.3
Oxacillin	β-Lactam	4	0	4	50.0	7	3	20	66.7
Penicillin G	β-Lactam	1	0	7	87.5	6	2	22	73.3
Rifampicin	Ansamycine	3	1	4	50.0	17	0	13	43.3
Synercid	Streptogramine	5	0	3	37.5	11	2	17	56.7
Teicoplanin	Glycopeptide	8	7	0	0.0	8	17	5	16.7
Tigecycline	Glycylcycline	4	0	4	50.0	9	1	20	66.7
Trimethoprim/ sulphamethoxazole	Dihdrofolatreductase/ sulfonamide	2	1	5	62.5	6	3	21	70.0
Vancomycin	Glycopeptide	8	0	0	0.0	17	9	4	13.3

S—susceptible; I—immediate; R—resistant; RR—resistance rate.

**Table 5:** PCR results for detection of resistance-associated genes of staphylococci.

Resistance-Associated Genes		*Staphylococcus aureus* (*n* = 8)		Non-*Staphylococcus aureus* (*n* = 30)	
		Detected (*n*)	%	Detected (*n*)	%
β-Lactam resistance	*mec*A	7	87.5	29	96.7
	*mec*B	0	0.0	0	0.0
	*mec*C	0	0.0	0	0.0
Penicillin resistance	*bla*Z	8	100	22	73.3
Linezolid resistance	*optr*A	4	50.0	3	10.0
	*val*S	8	100	9	30.0
	*cfr*	0	0.0	0	0.0
Erythromycin resistance	*erm*(B)	6	75.0	15	50.0
	*erm*(A)	2	25.0	1	3.33
	*erm*(C)	7	87.5	16	53.3
Vancomycin resistance	*van*A	0	0.0	2	6.7
	*van*B	0	0.0	9	30.0
	*van*C1	5	62.5	2	6.7
Macrolide resistance	*msr*C	6	75.0	4	13.3
Aminoglycoside resistance	*aac-aph*D	7	87.5	17	56.7
Tetracycline resistance	*tet*K	8	100	24	80.0
	*tet*M	2	25.0	4	13.3
	*tet*L	4	50.0	7	23.3
	*tet*S	0	0.0	3	10.0
	*tet*O	0	0.0	0	0.0

**Table 6:** Antibiotic resistance-associated genes in streptococci.

	Penicillin Resistance	Macrolide Resistance					Lincosamide Resistance		Aminoglycoside Resistance		Tetracycline Resistance				
	*bla*Z	*mef*A	*erm*(TR)	*erm*(C)	*erm*(B)	*erm*(A)	*lnu*A	*lnu*D	*aph*A-3	*aad*-6	*tet*S	*tet*K	*tet*L	*tet*M	*tet*O
*Streptococcus dysgalactiae* (*n* = 3)	3	0	0	3	3	3	3	0	2	0	0	0	3	3	0
*Streptococcus agalactiae* (*n* = 1)	1	0	0	1	1	0	1	0	0	0	0	1	1	1	0
*Streptococcus suis* (*n* = 1)	1	0	0	1	1	1	1	0	1	0	0	0	1	1	0
*Streptococcus gallolyticus* (*n* = 1)	1	0	0	1	1	0	1	0	1	1	1	1	1	1	0
